# Identification and phylogenetic analysis of the complete mitochondrial genome of Fuzhong buffalo (Artiodactyla: Bovidae)

**DOI:** 10.1080/23802359.2020.1714505

**Published:** 2020-01-20

**Authors:** Meng-Wei Li, Qian Lin, Yan-Zhou Wang, Jia-Xiang Huang, Hua-Jiao Qiu

**Affiliations:** a Chinese Academy of Agricultural Sciences and Guangxi Zhuang Nationality Autonomous Region, Buffalo Research Institute, Nanning, China;; b Chinese Academy of Agricultural Sciences, Institute of Bast Fiber Crops, Changsha, China

**Keywords:** Fuzhong buffalo, mitochondrial genome, phylogenetic analyses

## Abstract

Fuzhong buffalo (*Bubalus bubalis* Linnaeus, 1758 breed Fuzhong, FB) is one of the famous indigenous breeds of buffalo in China. It is the first time that the complete mitochondrial genome sequence of the FB was reported. The total length of the mtDNA is 16,363 bp, It contains the typical structure, including 22 transfer RNA genes, two ribosomal RNA genes, 13 protein-coding genes and one non-coding control region (D-loop region). The overall composition of the mtDNA was estimated to be 32.98% for A, 26.34% for T, 26.70% for C and 13.98% for G. Phylogenetic analyses using N-J computational algorithms showed that the analyzed 19 ruminantia species are divided into four major clades: Bovidae, Cervidae, Giraffidae and Atilocapridae. In addition, our work confirmed that FB and Murrah buffalo (MB) have a close genetic relationship with fellow tribal members Nili-Ravi buffalo and Mediterranean buffalo. Meanwhile, we also found that FB and MB have a highly similar genetic relationship.

Fuzhong buffalo (*Bubalus bubalis* Linnaeus, 1758 breed Fuzhong, FB) is one of the famous indigenous breeds of buffalo in China. In this study, we newly determined the complete mitochondrial genome of FB, and the specimens were collected from the adult individuals of FB at its culturing farm in Nanning city (22°90′29.27″N and 108°36′07.38″E), Guangxi Zhuang Nationality Autonomous Region, China on September 2019. And the specimens were stored at −80 °C in the National Buffalo Resources Specimen Library of China (Buffalo Research Institute, Chinese Academy of Agricultural Sciences and Guangxi Zhuang Nationality Autonomous Region, Nanning, China) with a catalog number of FB20190901. Total genomic DNA was extracted from the whole blood specimen of a single individual using the EasyPure Kit of Genomic DNA (Transgen Biotech, Beijing, China). Whole mitochondrial genome was amplified with 11 pairs of primers and sequenced by BioSune Biotech (Shanghai, China). DNA sequence was analyzed using DNAStar.Lasergene.v7.1 software (Madison, WI), tRNA Scan-SE1.21 software (Lowe and Eddy 1997) and DOGMA software (Wyman et al. 2004).

The total length of the mtDNA is 16,363 bp, It contains the typical structure, including 22 transfer RNA genes, two ribosomal RNA genes, 13 protein-coding genes and one non-coding control region (D-loop region) (GenBank accession No. MN756623). The overall composition of the mtDNA was estimated to be 32.98% for A, 26.34% for T, 26.70% for C and 13.98% for G, in the order A > C > T > G feature occurs in the FB. All the protein initiation codons are ATG, except for ND2, ND3 and ND5 are ATA. All these genes have 13 spaces and 11 overlaps both in the length of 1–40 bp. These genes had four types of termination codons, including TAA, TAG, AGA and an incomplete termination codon ‘T––’. ‘T––’ is the 5’ terminal of the adjacent gene (Anderson et al. 1981). Among 13 protein-coding genes, the longest one was ND5 gene (1821 bp), which was located between the tRNA^Leu^ and ND6, and the shortest one was ATPase8 gene (201 bp), which was located between the tRNA^Lys^ and ATPase6. The lengths of 12S rRNA and the 16S rRNA were 957 bp and 1569 bp. And deduced 22 tRNA genes were distributed in rRNA and protein-coding genes, ranging from 60 to 75 bp in size. The mitochondrial DNA D-loop region of the FB was located between tRNA^Pro^ and tRNA^Phe^ with a length of 929 bp.

Phylogenetic analysis was performed using the complete mitochondrial DNA sequences of 19 ruminantia species. Each of the sequence dataset was aligned by ClustalX (Thompson et al. 1997) and analyzed by neighbour-joining (N-J) in MEGA 4.0 (Tamura et al. 2007), and bootstrap analysis was performed with 100 replications. An N-J tree showed that the analyzed species are divided into four major clades (Figure 1). Bovidae makes up the first lineage, which is sister to the second group, Cervidae; Giraffidae forms the third group and is sister to Bovidae and Cervidae. The lineage consisting of these three groups in turn is sister to the fourth clade, Atilocapridae. In addition, our work confirmed that FB and Murrah buffalo (MB) have a close genetic relationship with fellow tribal members Nili-Ravi buffalo (NRB) and Mediterranean buffalo (MEB). Meanwhile, we also found that FB and MB have a highly similar genetic relationship.

**Figure 1.**
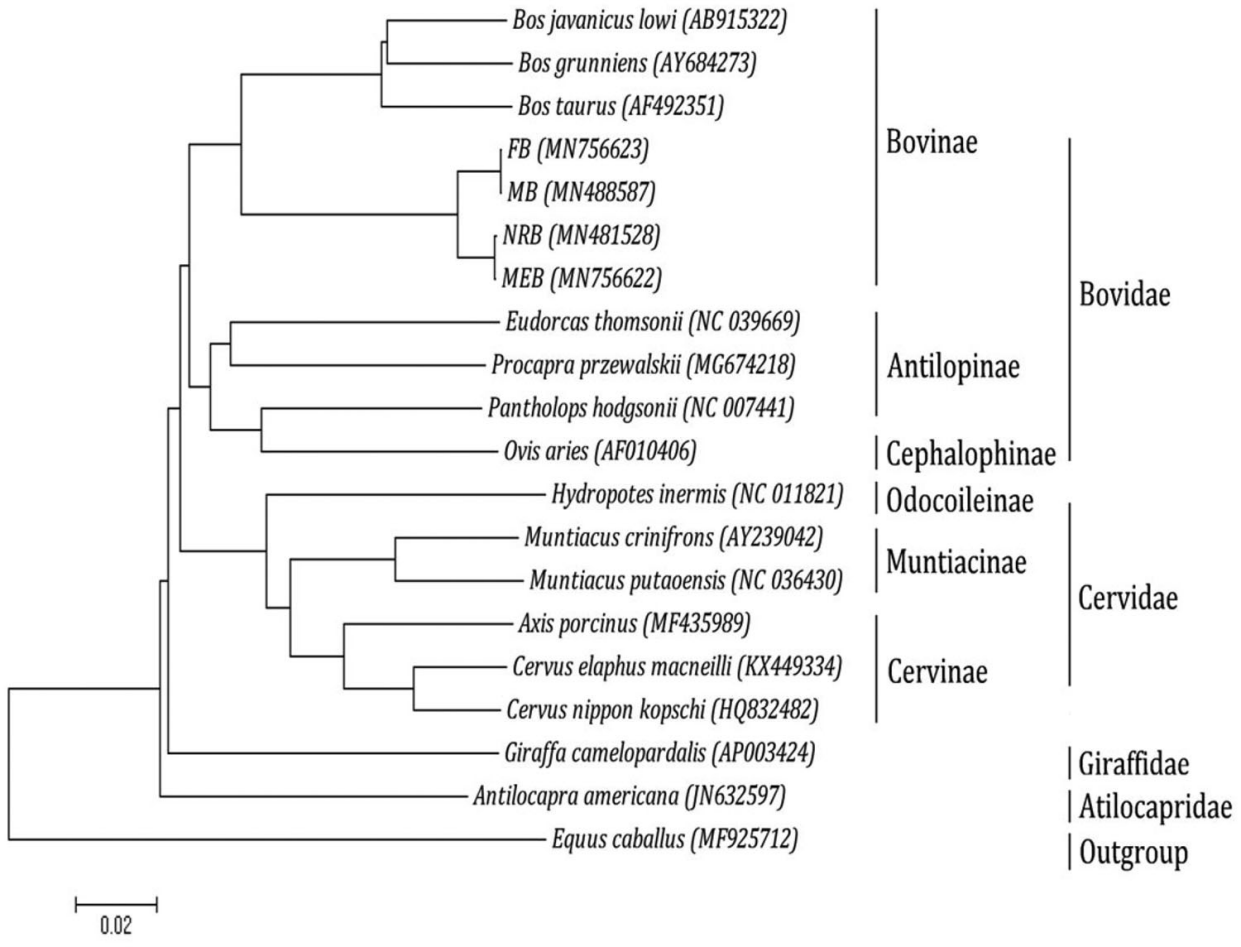
Phylogenetic analysis based on complete mitochondrial genome sequences. An N-J tree was built based on the phylogenetic analysis of 19 ruminantia species’ complete mitochondrial genomes. The mitochondrial genome sequences of the ruminantia species were obtained from the GenBank databases (Accession numbers have marked on the figure). Abbreviation of species indicates FB: Fuzhong buffalo; MB: Murrah buffalo; NRB: Nili-Ravi buffalo; MEB: Mediterranean buffalo.

## References

[CIT0001] Anderson S, Bankier AT, Barrell BG, de Bruijn MH, Coulson AR, Drouin J, Eperon IC, Nierlich DP, Roe BA, Sanger F, et al. 1981. Sequence and organization of the human mitochondrial genome. Nature. 290(5806):457–464. 7219534 10.1038/290457a0

[CIT0002] Lowe TM, Eddy SR. 1997. tRNAscan-SE: A program for improved detection of transfer RNA genes in genomic sequence. Nucleic Acids Res. 25(5):955–964. 9023104 10.1093/nar/25.5.955 PMC146525

[CIT0003] Tamura K, Dudley J, Nei M, Kumar S. 2007. MEGA4: molecular evolutionary genetics analysis (MEGA) software version 4.0. Mol Biol Evol. 24(8):1596–1599. 17488738 10.1093/molbev/msm092

[CIT0004] Thompson JD, Gibson TJ, Plewniak F. 1997. The Clustal-X Windows interface: Flexible strategies for multiple sequence alignment aided by quality analysis tools. Nucleic Acids Res. 25(24):4876–4882. 9396791 10.1093/nar/25.24.4876 PMC147148

[CIT0005] Wyman SK, Jansen RK, Boore JL. 2004. Automatic annotation of organellar genomes with DOGMA. Bioinformatics. 20(17):3252–3255. 15180927 10.1093/bioinformatics/bth352

